# Grand challenge in antibiotic pharmacology: A major step toward tailored antimicrobial treatment in very complex clinical scenarios of infectious risk management

**DOI:** 10.3389/frabi.2022.1016760

**Published:** 2022-09-20

**Authors:** Federico Pea

**Affiliations:** ^1^Department of Medical and Surgical Sciences, Alma Mater Studiorum, University of Bologna, Bologna, Italy; ^2^Clinical Pharmacology Unit, Department for Integrated Infectious Risk Management, Istituto di Ricovero e Cura a Carattere Scientifico (IRCCS) Azienda Ospedaliero-Universitaria di Bologna, Bologna, Italy

**Keywords:** clinical pharmacology and therapeutics, pharmacokinetic variability, PK/PD modelling, TDM (therapeutic drug monitoring), personalized and precision medicine (PPM)

## Appropriate antibiotic use: Where we are and where we have to go

Morbidity and mortality associated with bacterial infections from multi drug-resistant pathogens is a major health problem among hospitalized patients. This is especially true among those belonging to some special populations, such as the critically ill patients admitted to the intensive care unit (ICU). Optimizing the use of antimicrobial drugs may make possible to effectively counteract this problem by either maximizing the probability of therapeutic success or allowing microbiological eradication (Roberts et al., 2014). Additionally, achieving this latter goal may be especially fundamental for limiting the development of bacterial resistance to antibiotics (Roberts et al., 2014) (1).

## The concept of tailored antimicrobial therapy

Personalization of antimicrobial therapy (the so-called “tailored antimicrobial therapy”) has gained considerable scientific evidence in recent years as a useful “antimicrobial stewardship” tool for counteracting the epidemiological context of infections caused by multi-drug resistant pathogens (Pea and Viale, 2009; Roberts et al., 2012; Roberts et al., 2014). Unfortunately, prevalence and incidence of multi-drug resistant bacteria, especially among Gram-negatives, is showing an ever growing increase in many countries around the world. This challenge offers major concerns in managing properly the infectious risk in highly complex settings of special patient populations. In this regard, data coming from the European Center for Disease Prevention and Control (ECDC) showed that in several European countries the 2019-2020 resistance rates of *Pseudomonas aeruginosa*, of Enterobacterales and of *Acinetobacter baumannii* to carbapenems may be higher than 20-50% (European Food Safety Authority and European Centre for Disease Prevention and Control, 2022). This means that these fundamental therapeutic weapons are progressively losing their role against the most challenging Gram-negatives.

Inappropriate use of antibiotics often results in the possibility of causing inadequate therapeutic exposure. Under-exposure can cause therapeutic failure (Pea and Viale, 2009) and may represent a factor of selective pressure in promoting the onset of bacterial resistance (Roberts et al., 2014; Sumi et al., 2019; Gatti and Pea, 2021a; Gatti et al., 2021). The optimisation or lack optimisation of antibiotic concentrations at the site of the infectious lesion may be a contributor to success or failure and the emergence of resistance (Pea and Viale, 2009; Roberts et al., 2014). On the other hand, it should also not be overlooked that overexposure may increase the toxicity risk in some patient settings, as it may occur particularly in the fragile elderly population (Pea, 2015).

## Clinical pharmacology of tailored antimicrobial therapy

Clinical pharmacology of tailored antimicrobial therapy has the purpose of choosing the correct antibiotic dosage for each individual patient, possibly in real time. It should be based on the results of therapeutic drug monitoring (TDM) of antibiotic concentrations and on their expert interpretation in relation to the *in vitro* susceptibility of the bacterial clinical isolate (MIC), the site of infection, the patient’s pathophysiological features and the co-treatments responsible for eventual drug-drug interactions (Gatti et al., 2022).

This approach was just proven to be particularly useful in the critically ill patients, which are a population at very high risk of inadequate antibiotic exposure during treatment with standard dosages (Roberts et al., 2012; Roberts et al., 2014). In this regard, several international scientific societies have recently implemented guidelines with specific recommendations aimed at such an approach in the critically ill patients. Just to mention some of these, I can cite those co-authored by the French Society of Pharmacology and Therapeutics (Société Française de Pharmacologie et Thérapeutique-SFPT) and the French Society of Anaesthesia and Intensive Care Medicine (Société Française d’Anesthésie et Réanimation-SFAR) for optimizing treatment with beta-lactam antibiotics (Guilhaumou et al., 2019); those of the Surviving Sepsis Campaign for managing sepsis and septic shock (Rhodes et al., 2017), and those co-authored by the Infectious Diseases Society of America and the American Thoracic Society for optimizing treatment of hospital-acquired and ventilator-associated pneumonia (Kalil et al., 2016). In addition, a recent position paper co-authored by a multidisciplinary panel of authoritative international researchers with expertise in the field of antimicrobial TDM in critically ill adult patients has strongly reiterated that this approach should be considered the only effective and safe way for optimizing the management of antibiotic therapy in this setting (Abdul-Aziz et al., 2020). Besides, it should not be overlooked that TDM is usually performed in serum/plasma, and this could mean that the quality of the prediction of outcome could be lower in case of deep-seated infections compared to bloodstream infections.

## Challenges in dealing with tailored antimicrobial therapy in the critically ill patients

The critically ill patients are the special population in which the issue of tailoring antimicrobial therapy was most frequently investigated up to date. Why this approach started from the critically ill patients? Critically ill patients are often affected by severe infections (sepsis) coupled with peculiar pathophysiological features that may alter the pharmacokinetic behavior of antibiotics (**Table 1**) (Pea et al., 2005; Pea and Viale, 2009; Roberts et al., 2012; Roberts et al., 2014; Blot et al., 2014). The mortality rate of septic patients is non-negligible, and this is probably also because of the increasingly prevalent antimicrobial resistance. Therapeutic success is associated with early diagnosis and timely implementation of appropriate antimicrobial treatment, which should be based on both appropriate bacterial coverage and correct dosing regimens (Pea and Viale, 2006; Roberts et al., 2014; Adembri et al., 2020). Optimized plasma exposure to antimicrobials was just associated with better clinical and/or microbiological outcome in some studies (Roberts et al., 2014; Economou et al., 2017; Gatti et al., 2021). Some major pathophysiological changes may cause inadequate exposure to antibiotics in septic patients (Pea et al., 2005; Roberts et al., 2014; Blot et al., 2014). The following are some relevant examples. The sepsis-related tissue capillary leakage coupled with the fluid load needed for maintaining volemia, which may cause an increase in volume of distribution of antibiotics (Roberts et al., 2012; Roberts et al., 2014). The hypoalbuminemic status, which may favour an increased renal elimination of highly protein-bound antimicrobials (Pea and Viale, 2009). The status of transient acute kidney injury (AKI), a rather frequent temporary condition associated with sepsis, which may favour antibiotic under-exposure whenever timely recovery of renal function within the first 48 hours is not promptly addressed by increasing antimicrobial dosing (Sumi et al., 2019; Crass et al., 2019). The application of continuous renal replacement therapies (CRRTs), which may have a direct influence on antimicrobial elimination and may represent extremely complex scenarios for choosing appropriate antimicrobial dosages (Bugge, 2004; Jamal et al., 2015; Economou et al., 2017; Sumi et al., 2019; Crass et al., 2019; Hoff et al., 2020; Li et al., 2020; Roberts et al., 2020; Gatti and Pea, 2021b). The so-called augmented renal clearance (ARC), which may cause faster elimination of renally cleared antibiotics with the need of more aggressive dosing schedule regimens (Hefny et al., 2022; Silva et al., 2022).

**Table 1:** Challenges in dealing with antimicrobial tailored therapy in some special patient populations and possible solutions.

Special population	Key features	Solutions
Critically ill patients	* Sepsis-related tissue capillary leakage * Fluid load * Hypoalbuminemia * Transient AKI * Application of CRRT * ARC	* TDM-guided antimicrobial dosing * Specific population PK/PD studies
Oncohematological patients	* Hypoalbuminemia * ARC	* TDM-guided antimicrobial dosing * Specific population PK/PD studies
Pediatric critically ill patients	* Age-related variations of the intra/extra-cellular water distribution * Age-related changes in renal function * Neonatal sepsis * Oncohematological malignancies and/or hematopoietic stem cell transplantation	* TDM-guided antimicrobial dosing * Specific population PK/PD studies
Frail elderly patients	* Decline in functional reserves * Various degrees of renal dysfunction * Need of polypharmacy or hyper-polypharmacy	* TDM-guided antimicrobial dosing * Specific population PK/PD studies
Patients with subacute-chronic infections	* Limited antimicrobial penetration into deep-tissues * Development of biofilm in presence of prosthetic material	* TDM-guided antimicrobial dosing * Specific population PK/PD studies
Patients with post-neurosurgical infections	* ARC in patients with subarachnoid hemorrhage and/or intracerebral hemorrhage	* TDM-guided antimicrobial dosing * Specific population PK/PD studies
Extensive burn patients	* Massive fluid support in the early post-event phase * Massive edema * ARC * Hypoalbuminemia * Multiple sepsis-related organ failure in the late stages	* TDM-guided antimicrobial dosing * Specific population PK/PD studies

AKI, Acute kidney injury; ARC, augmented renal clearance; CRRT, continuous renal replacement therapy; PK/PD, pharmacokinetic/pharmacodynamic.

## Challenges in dealing with tailored antimicrobial therapy in other special patient populations

In addition to the critically ill patients, it should be mentioned that there are several other special patient populations that may greatly benefit from the approach of tailored antimicrobial therapy. The following is a non exhaustive list of examples for future studies.

## Challenges in dealing with tailored antimicrobial therapy in oncohematological patients

Oncohematological patients are at high risk of developing bacterial infections during febrile neutropenia (Guarana et al., 2019). The need of optimizing antimicrobial dosages in this population is related to some peculiar patophysiological characteristics (**Table 1**). ARC is highly prevelent in patients with febrile netropenia (Cook and Hatton-Kolpek, 2019; Cojutti et al., 2020) and may promote faster elimination of hydrophilic antimicrobials (Cojutti et al., 2020). Hypoalbuminemia is a rather frequent occurrence in this setting and may promote fluid extravasation with increased volume of distribution of hydrophilic antibiotics and at the same time under-exposure of renally cleared antibiotics used at standard dosing regimens (Cojutti et al., 2020).

## Challenges in dealing with tailored antimicrobial therapy in the pediatric critically ill patients

Unfortunately, performing dose finding studies with antimicrobials in the specific setting of pediatric critically ill patients is quite challenging for a number of reasons. This means that most antimicrobials have no specific dosing regimens validated in this setting. Antimicrobial doses are frequently extrapolated by those used in adults and adjusted by weight or by body surface area. This approach is somewhat simplistic and can often cause inadequate exposures in the pediatric patient (Ritz et al., 2016). The major physiological variations of the intra/extra-cellular water distribution and/or of the different degree of renal function associated with the various developmental ages may impact on the pharmacokinetic behavior of antimicrobials (**Table 1**) (Ritz et al., 2016). Additionally, the presence of some underlying pathophysiological conditions, such as neonatal sepsis, oncohematological malignancies and/or hematopoietic stem cell transplantation, may furtherly exacerbate this issue (Dvorak et al., 2012; Downes et al., 2014; Messinger et al., 2017; Kyriakidis et al., 2017; Kebudi and Kizilocak, 2018; Czyżewski et al., 2019).

## Challenges in dealing with tailored antimicrobial therapy in the frail elderly patients

The need to customize antimicrobial treatment may be especially relevant in frail elderly patients, in whom the right balance between efficacy, safety and tolerability could be very difficult to be addressed (**Table 1**) (Pea, 2015). Frailty is a condition essentially related to a decline in functional reserves and is considered an important risk factor for adverse effects among the elderly. Frailty per se may affect antimicrobial exposure as it may be associated to various degrees of renal dysfunction. Additionally, the frequent need of polypharmacy or hyper-polypharmacy in frail elderly patients with multiple co-morbidities may lead to major drug-drug interactions responsible for altered antimicrobial exposure (Pea, 2015; Pea, 2018). This may expose the frail elderly with severe infections to an increased risk of overexposure and toxicity when are treated with standard doses of antibiotics. That is why nowadays specific studies are increasingly claimed for customizing properly drug dosage in this special population (Cojutti et al., 2021).

## Challenges in dealing with tailored antimicrobial therapy in patients with subacute and chronic bacterial infections

Subacute and chronic bacterial infections, like osteomyelitis, spondylodiscitis, prosthetic joint infections, vascular-prosthetic infections and post-traumatic infections are characterized by a high burden of management complexity, care and costs for the health system (**Table 1**) (Almangour and Alhifany, 2020). Antimicrobial penetration into bones or other types of deep-tissues may be limited (Thabit et al., 2019; Almangour and Alhifany, 2020). The production of biofilm by various microbial pathogens may furtherly worsen the situation in presence of prosthetic material by favoring bacterial sequestration (Thabit et al., 2019; Almangour and Alhifany, 2020). Consequently, in these cases antibiotic treatment should last for several weeks or even months and dealing properly with the correct dosing adjustments over time could be challenging (Thabit et al., 2019). Specific studies addressing the issue of proper dosing regimens could be extremely relevant in reducing the risk of therapeutic failure and/or relapse in this setting.

## Challenges in dealing with tailored antimicrobial therapy in patients with post-neurosurgical infections

Neurosurgical patients may often need temporary implantation of external ventricular drainages (EVDs) for monitoring intracranial pressure, as well as for CSF outflow in pathological conditions such as acute hydrocephalus, cerebral hemorrhage and/or traumatic brain damage (Beer et al., 2008). The use of EVDs may be associated with an increased risk of bacterial ventriculitis, which may lead to increased morbidity, mortality, prolonged hospitalization, and cost increase (Beer et al., 2008). Neurosurgical patients may often suffer from pathophysiological alterations that can hinder the achievement of effective concentrations of antibiotics at the infection site when using standard doses (**Table 1**) (Lonsdale et al., 2013). Noteworthy, it was recently shown that the prevalence of ARC in patients with subarachnoid hemorrhage and/or intracerebral hemorrhage may be very high, ranging from 80 to 100% (Morbitzer et al., 2019). Consequently, specific studies addressing the issue of tailored antimicrobial therapy in this special population are urgently needed (Morbitzer et al., 2016).

## Challenges in dealing with tailored antimicrobial therapy in patient with extensive burns

Patients suffering from major burns involving more than 30% of the total body surface area represent a special and unique subpopulation of critically ill patients. Antibiotics are widely used to treat infectious complications that could by the consequence of major skin lesions, of prolonged use of intravascular devices, and/or of long-lasting hospitalization (Udy et al., 2018). Patients with extensive burns may present several pathophysiological conditions that may alter exposure to antibiotics when used at standard doses (**Table 1**). In this regard, massive fluid support in the early post-event phase, massive edema, ARC, hypoalbuminemia, and sepsis-related multiple organ failure in the late stages are important examples (Udy et al., 2018). Specific studies should be carried out for properly dealing with all of these issues in this special patient population.

## Future steps in antibiotic pharmacology

Nowadays, the role of real-time TDM-guided antimicrobial treatment is consistently increasing in the setting of critically ill patients (Abdul-Aziz et al., 2020; Gatti et al., 2022). This approach would reveal helpful in dealing with personalization of antimicrobial treatment also, but not only, for all of the other special patient populations that I mentioned in this article.

Another important step forward could be represented by the increasingly use of microdialysis. This tecnique may allow measuring antimicrobial concentrations directly at the infection site, and would provide important additional knowledge in case of deep-seated infections, even if some methodological limits nowadays may affect its reliability (Jager et al., 2019).

Finally, pharmacometrics may represent another important tool in properly dealing with all of the challenging situations concerning tailored treatment with antibiotics in special patient populations. Pharmacometrics may quantify adequately information on drugs, diseases and pathophysiological features. It may allow to define the relationship existing between drug exposure, namely pharmacokinetics (PK), and drug response, namely pharmacodynamics (PD), in terms of desired and undesirable effects. This approach is based on modelling specific features of each individual patients, so that it may be very helpful in addressing proper use of drugs. Implementing population PK and PK/PD models of antimicrobials in each single special patient population may allow identifying the most appropriate dosage for granting optimized tailored treatment in each of the different scenarios (Pereira et al., 2022).

## Expectations of the Pharmacology Section of Frontiers in Antibiotics

Overall, the Pharmacology Section of Frontiers in Antibiotics will very well welcome all the studies based on clinical pharmacology of antimicrobials that will be conducted by means of real-time TDM, population PK and/or PK/PD modelling and/or by any other kind of approach focused on providing major contributes and important advances in understanding how to maximize effectiveness with antimicrobials in each of the very complex clinical scenarios of infectious risk management that may characterize different types of special patient populations.

## Author contributions

The author confirms being the sole contributor of this work and approved it for publication.

## Conflict of interest

The author declares that the research was conducted in the absence of any commercial or financial relationships that could be construed as a potential conflict of interest.

## Publisher’s note

All claims expressed in this article are solely those of the authors and do not necessarily represent those of their affiliated organizations, or those of the publisher, the editors and the reviewers. Any product that may be evaluated in this article, or claim that may be made by its manufacturer, is not guaranteed or endorsed by the publisher.
